# Investigation of Parasitic Ticks and the Potential Tick-Borne Pathogenic Fungi in Yunnan Province, Southwest China

**DOI:** 10.3390/microorganisms14091911

**Published:** 2026-08-28

**Authors:** Meng-Ling Deng, Yan Zhang, Man-Li Jiang, Tong Zhang, Jun Ma, Jian-Fa Yang, Feng-Cai Zou, Jun-Jun He, Lu-Yang Wang

**Affiliations:** 1 The Yunnan Key Laboratory of Veterinary Etiological Biology, College of Veterinary Medicine, Yunnan Agricultural University, Kunming 650201, China; 2 College of Bioengineering, Sichuan Water Conservancy Vocational College, Chengdu 611130, China

**Keywords:** ticks, pathogenic fungi, Yunnan Province

## Abstract

Ticks are hematophagous arthropods that parasitize livestock, humans, and wild animals, posing serious threats to public health worldwide. Diverse pathogens have been discovered in ticks, including viruses, bacteria, protozoa, and filarial nematodes; however, ticks also harbor a variety of fungal microbes, which remain largely unexplored. In this study, we isolated and identified culturable fungi associated with ticks collected on cattle and goats in Yunnan Province, China. Of the 1730 ticks obtained, 1690 were identified as *Rhipicephalus microplus*, and only 40 as *Haemaphysalis longicornis* via morphology and 16S rRNA gene analysis. A total of 90 fungal strains were isolated from the ticks and identified by morphological examination and ITS sequencing, representing 25 species across 20 genera, with *Fusarium* being the most prevalent. Among these isolates, *Fusarium verticillioides* (19 isolates) poses a threat to grains, animals, and humans; *Cladosporium cladosporioides* (six isolates) and *Sarocladium zeae* (one isolate) are primarily pathogenic to plants, while *Schizophyllum commune* (eight isolates), *Rhizopus arrhizus* (two isolates), *Diaporthe phaseolorum* (two isolates), and *Purpureocillium lilacinum* (two isolates) are recognized animal-associated pathogens. The diversity of isolated fungi with documented pathogenicity suggests that ticks may serve as potential vectors for fungal transmission, raising concerns about their role in cross-kingdom disease spread across agriculture and livestock. Furthermore, fungi with entomopathogenic potential, such as *P. lilacinum*, offer valuable resources for developing biocontrol agents against ticks. Our findings highlight the dual role of ticks as both potential vectors of phytopathogenic fungi and reservoirs of entomopathogenic biocontrol agents. This necessitates a paradigm shift in tick surveillance, integrating mycological monitoring to mitigate agricultural losses and exploring tick-borne fungi for sustainable vector control strategies.

## 1. Introduction

Ticks are the most common ectoparasites found on different vertebrates in various stages; tick infestation poses a risk to the world’s population [1]. In China, more than 120 tick species have been reported, including 14 species belonging to the *Argasidae* family and 111 species from the Ixodidae family [2]. Ticks are globally distributed, temporary blood-sucking arthropods that can infest a wide range of animals and humans [3,4]. They transmit diverse bacterial, fungal, viral, and parasitic pathogens to both humans and animals [5,6], causing major public health problems and considerable socioeconomic losses to the livestock industry, particularly in tropical and subtropical regions [7,8]. Approximately 80% of the global cattle population is infested with ticks and tick-borne pathogens, leading to direct production losses and transmission of transboundary cattle diseases. Additionally, ticks harbor a variety of non-pathogenic microbes, including commensal and mutualistic species [9,10,11,12]. Some of these microbes can modulate the tick’s immune system, thereby influencing its resistance to pathogens, and others may play a critical role in tick development and survival, for example, by synthesizing essential nutrients [13]. Beyond their internal microbes, ticks carry a taxonomically diverse cuticular microbiota. Yet, how this external community affects ticks’ ecological role remains largely unknown.

The advent of next-generation sequencing technologies has led to a growing interest in the tick microbiome, resulting in the identification of an increasing number of tick-associated microorganisms. The composition of these microbial communities is highly variable, and their richness and diversity are largely influenced by environmental factors. Specifically, the external microbiota of ticks may originate from two primary sources: acquisition from the host skin microbiome during blood feeding, and uptake from environmental reservoirs, such as soil, plants, or vertebrate nests and burrows, during off-host periods [14,15,16]. Among these microorganisms, some may persist as long-term symbionts exerting little impact on ticks, whereas others may act as opportunistic pathogens that adversely affect tick survival [9,17].

In southwest China, Yunnan Province covers a vast area characterized by diverse climates, diverse geographical landscapes, and rich biodiversity; it provides ideal habitats for ticks, making it a hotspot for tick species diversity (47 species belonging to seven genera and two families, accounting for approximately one-third of tick species documented in China) and a high-risk zone for tick-borne diseases [18]. Among the various livestock species in Yunnan, cattle and goats are particularly important to local agriculture and the rural economy. The close contact between these animals and tick habitats facilitates frequent tick infestations, potentially exposing both livestock and humans to tick-borne pathogens. While most tick-associated microbiological studies have focused on bacterial communities and viral pathogens, tick-borne fungi have received considerably less attention. Fungi associated with ticks may play diverse roles, ranging from mutualistic symbionts that affect tick physiology and survival to opportunistic pathogens that could influence tick vector competence. Furthermore, some tick-associated fungi may produce bioactive metabolites with potential biotechnological or pharmaceutical applications. However, the culturable fungal communities carried by ticks infesting cattle and goats in Yunnan remain largely unexplored. In this study, we collected ectoparasitic ticks from livestock across seven different regions in Yunnan to investigate the parasitic ticks and the potential tick-borne pathogenic fungi. The findings are expected to provide a foundation for understanding the ecological roles of tick-associated fungi and their potential implications for tick control and public health.

## 2. Materials and Methods

### 2.1. Sample Sources and Morphological Identification of Ticks

**Figure 1:**
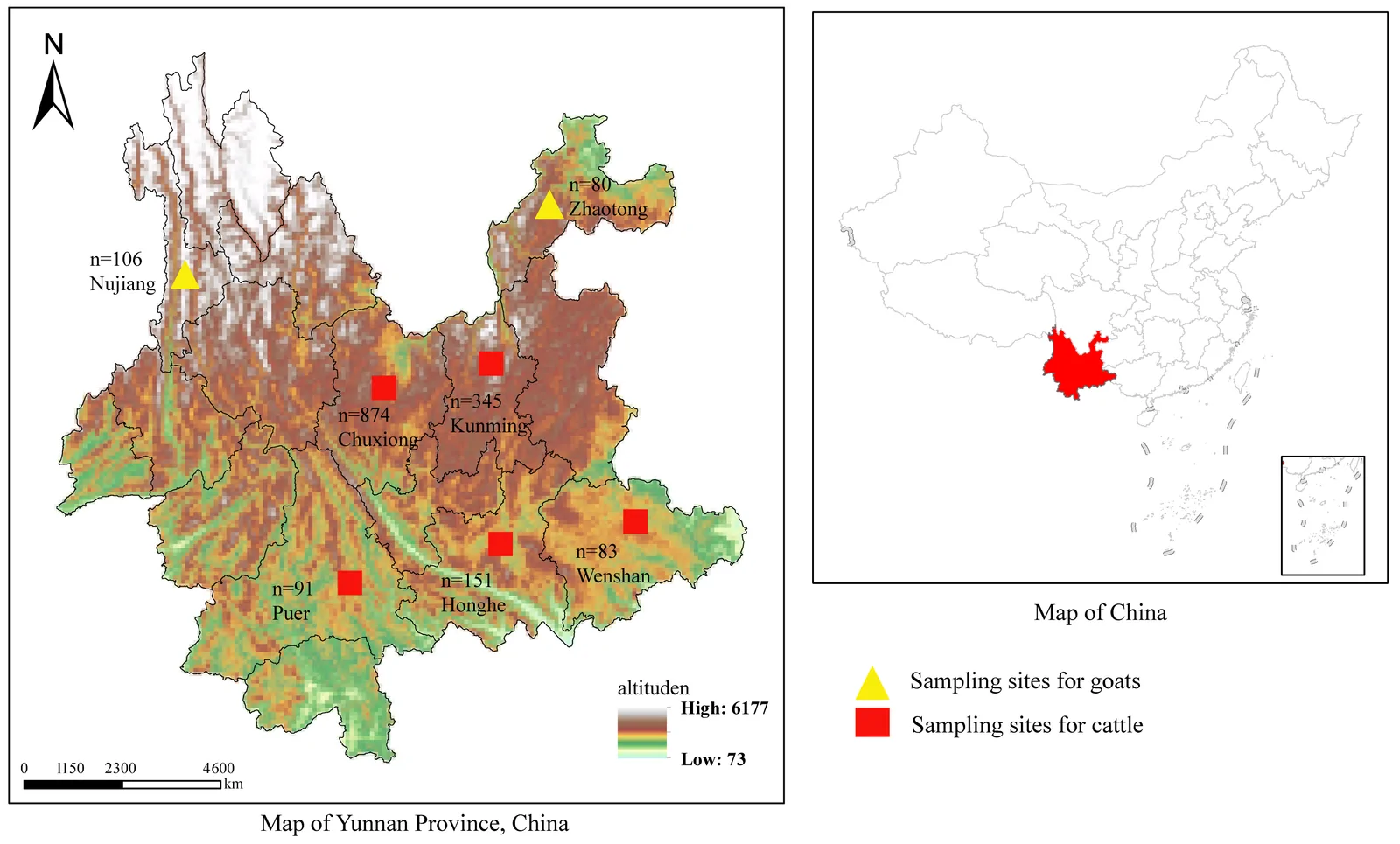
Geographic distribution of tick sampling sites in Yunnan Province, China. Yellow triangles: goat sampling sites; red squares: cattle sampling sites. Values adjacent to sites (*n*) indicate tick counts per locality. Background color gradient represents altitude (m), from 73 m (low) to 6177 m (high).

Geographical, climatic, and ecological conditions were considered, and samples were collected in seven regions of Yunnan Province, including Chuxiong Yi Autonomous Prefecture (*n* = 874), Kunming (*n* = 345), Honghe Hani and Yi Autonomous Prefecture (*n* = 151), Nujiang Lisu Autonomous Prefecture (*n* = 106), Pu’er (*n* = 91), Wenshan Zhuang and Miao Autonomous Prefecture (*n* = 83), and Zhaotong (*n* = 80). Sampling was conducted from April 2023 to September 2024. Tick samples were collected from cattle and goats in each region, as these livestock are commonly infested by ticks and widely distributed throughout Yunnan; the numbers of ticks collected from each region and host species are summarized in Figure 1. A total of 1730 adult tick specimens were obtained and individually stored in sterile tubes at –20 °C; larvae were not included in this study. Morphological identification was conducted using stereomicroscopes based on standard taxonomic keys (such as basis capituli, length and shape of palpi, shape of scutum, coxal spur, anal groove, porose areas, spiracular plate, eyes, etc.) and descriptions from relevant studies [19,20].

### 2.2. Molecular Identification of Ticks

Each tick was surface-sterilized by sequential immersion in 70% (*v*/*v*) ethanol for 1 min, 2% (*v*/*v*) sodium hypochlorite for 3 min, and finally rinsed three times with sterile distilled water to remove residual sterilant [21]. A whole tick body was longitudinally cut into two equal parts with a sterile blade. One half was used for culturing fungi, and the other for DNA extraction was individually crushed using a pestle, then placed into a sterilized centrifuge tube and homogenized in 400 μL of sterile water. A total of 200 μL of the homogenized solution was used for the extraction of tick genomic DNA, and the DNA was extracted using a DNeasy Blood and Tissue Kit (TIAGEN, Beijing, China) according to the manufacturer’s instructions. A 460 bp fragment of tick mitochondrial 16S rDNA gene was amplified by PCR and sequenced using the universal primers 16SF (5′-CTGCTCAATGATTTTTTAAATTGCTGTGG-3′) and 16SR (5′-CCGGTCTGAACTCAGATCAAGT-3′) [22]. PCR was performed in a total of 25 μL, containing 12.5 μL 2 × Taq mix (Sangon Biotech Co., Ltd., Shanghai, China), 10 μM of each primer, 2 μL DNA template, and ddH_2_O. The PCR reaction system was as follows: 95 °C for 5 min, 35 cycles of 95 °C for 30 s, 54 °C for 30 s, 72 °C for 50 s, and a final extension at 72 °C for 8 min. Following visualization via electrophoresis in 1% agarose gel, positive PCR products were sent to Sangon Biotech Co., Ltd. (Shanghai, China).

### 2.3. Isolation and Morphological Observation of Fungal Isolates

Tick samples used for culturing were individually crushed using a pestle, then homogenized in sterile tubes containing 400 μL of Potato Dextrose Broth (PDB) growth medium. Each tick was individually homogenized, and up to five homogenates from different ticks were cultured on a single Petri dish in a cross-shaped pattern, with each culture spot separated by at least 24 mm. Then, the medium was placed in a 28 °C constant-temperature humidified fungal incubator for cultivation. After 48 h of inoculation, the growth of colonies was observed daily, selecting single-colony hyphae from the colonies that appeared and transferring them to fresh Potato Dextrose Agar (PDA) medium for purification. If morphologically distinct colonies appeared on the same plate, they were repeatedly subcultured until a pure culture was obtained.. To monitor potential contamination introduced during sample processing, sterile water rinse (obtained by rinsing surface-sterilized ticks) and uninoculated media were included as negative controls throughout the experiment. No fungal growth was observed in any of these controls, confirming that the fungal isolates originated from internal tick tissues rather than from surface or environmental contamination.

The PDA medium was inoculated with isolates and incubated at 28 °C for 7 d in complete darkness. The morphology and color of fungal colonies and the density of hyphae were observed on PDA. Microscopic features, including the color and morphology of the conidiophores and conidia, were observed using a biological microscope (BM2000, Nanjing MDS Instrument Co., Ltd., Nanjing, China).

### 2.4. Molecular Identification of Fungal Isolates

The genomic DNA was extracted from fungal isolates following the protocol [23], and the DNA was stored at −20 °C for polymerase chain reaction (PCR) amplification. The rDNA region of internal transcribed spacer (ITS) was amplified using the primer pair ITS1 and ITS4 [24]. PCR reaction was conducted in a 25 μL mixture consisting of 12.5 μL 2 × taq mix, 1 μm of each primer, and 50 ng template DNA. The PCR conditions were as follows: 95 °C for 5 min, 35 cycles of 94 °C for 45 s, 52 °C for 30 s, and 72 °C for 3 min, and the final extension was 72 °C for 15 min. The positive samples were sequenced at Sangon Biotech Co., Ltd. (Shanghai, China), and the sequences were obtained and deposited in GenBank.

### 2.5. Phylogenetic Analysis

To accurately determine the taxonomic placement of ticks and fungal isolates, a comprehensive phylogenetic analysis was conducted. Sequences were trimmed and low-quality ends removed with Chromas Lite v2.6 (Technelysium Pty Ltd.). Preliminary identification of closely related species was compared against the NCBI nucleotide database by Basic Local Alignment Search Tools (BLAST; https://blast.ncbi.nlm.nih.gov/blast/Blast.cgi, accessed on 18 December 2025). Fungal taxonomic assignment was performed based on the following criteria: sequences with ≥99% identity to reference sequences were assigned to the species level; those with 97–99% were assigned to the genus level; and those with <97% identity were recorded as unidentified. Phylogenetic analyses were performed using the 16S rRNA gene sequences for ticks and ITS gene sequences for fungi obtained in this study, along with reference sequences retrieved from GenBank. Multiple alignments were generated with MEGA 11 using the ClustalW algorithm under default parameters. For tick identification, neighbor-joining (NJ) trees were constructed using the Kimura 2-parameter model, with nodal support evaluated using 1000 bootstrap replicates. For fungal identification, maximum likelihood (ML) trees were constructed using the p-distance model, and nodal support was assessed with 1000 bootstrap replicates.

## 3. Results

### 3.1. Morphology and Molecular Identification of Ticks

**Figure 2:**
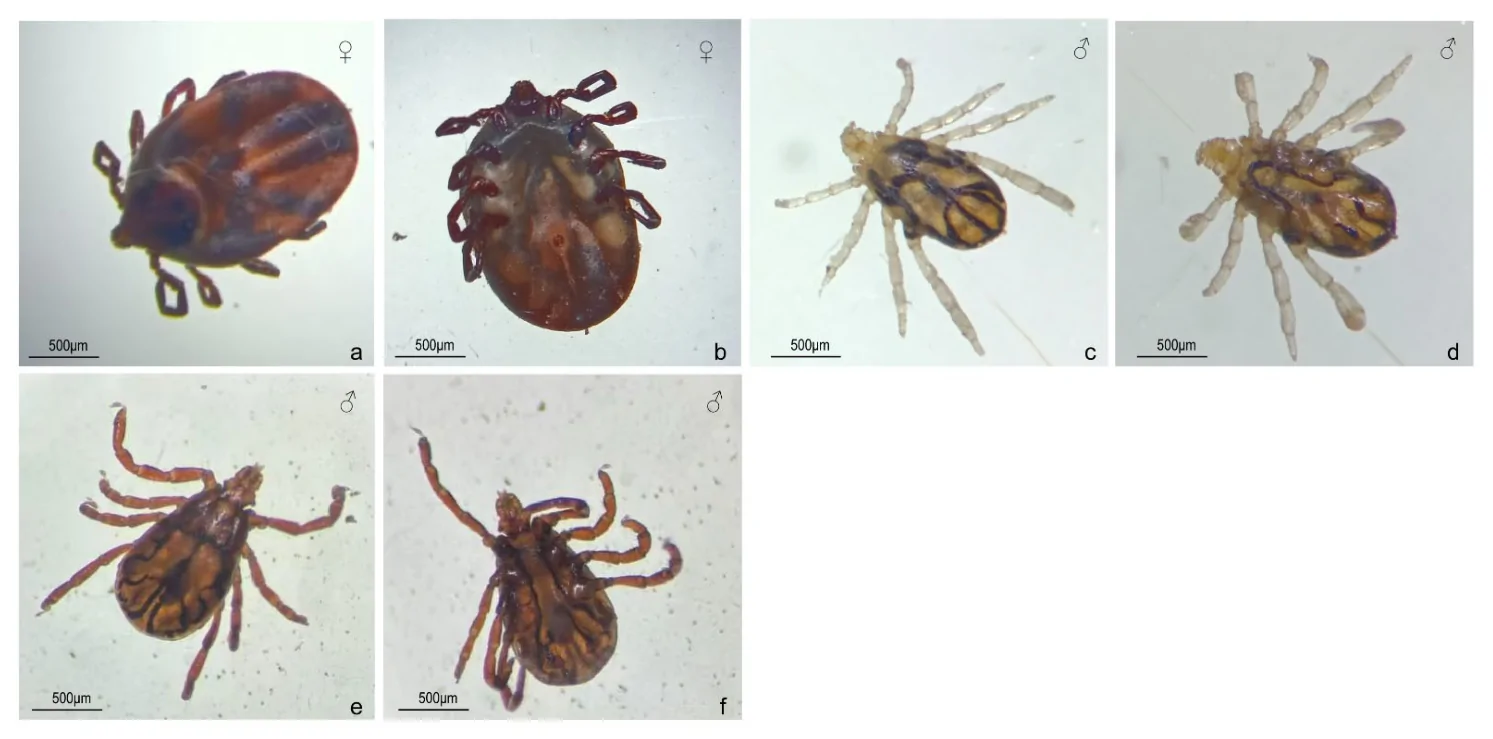
Morphological features of ticks collected from cattle and goats in Yunnan Province. *R. microplus* female (♀) and male (♂) specimens with ventral (**a**,**c**) and dorsal surface (**b**,**d**); *H. longicornis* male with ventral (**e**) and dorsal surface (**f**). (**a**): male *R. microplus* ventral surface; (**b**). male *R. microplus* dorsal surface; (**c**). male *H. longicornis* ventral surface; (**d**). male *H. longicornis* dorsal surface.

A total of 1730 adult ticks, including females and males, were collected from cattle (*n* = 1544) and goats (*n* = 186). All samples underwent morphological identification; two species belonging to two genera were identified, including one species from *Rhipicephalus* (*Rhipicephalus microplus*, *R. microplus*) and one from *Haemaphysalis* (*Haemaphysalis longicornis*, *H. longicornis*) (Figure 2). Among these, *R. microplus* was found to be widely distributed in Yunnan Province, being detected in all seven investigated regions; notably, *H. longicornis* was exclusively collected in Zhaotong. Additionally, we found that *R. microplus* (*n* = 1690) was the predominant species infesting cattle and goats in Yunnan Province, accounting for 97.69%; only 40 individuals of *H. longicornis* were detected.

**Figure 3:**
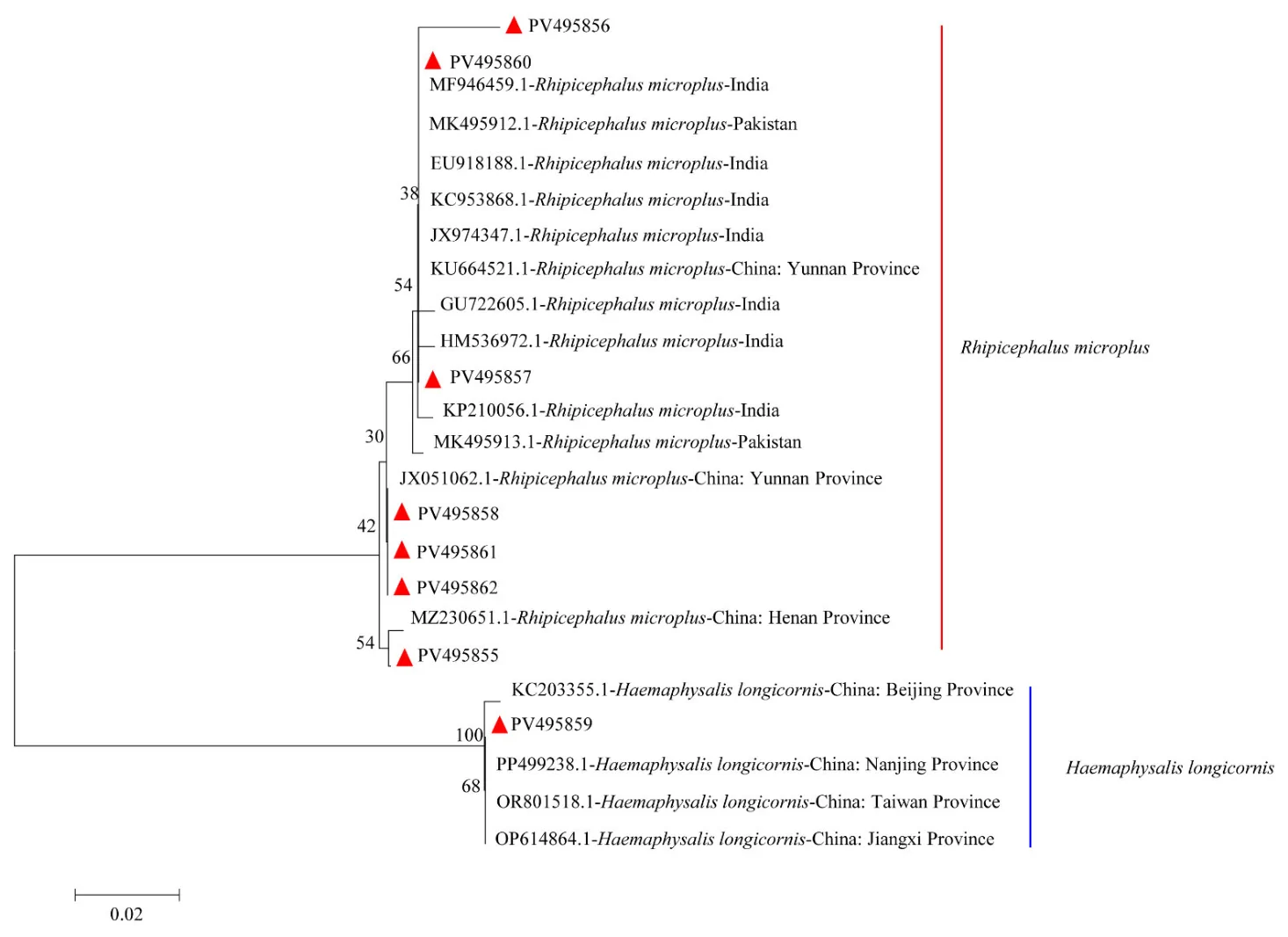
Phylogenetic tree based on tick 16S rRNA gene sequences. Evolutionary distances were calculated using the Kimura 2-parameter model, and nodal support was assessed with bootstrap analysis (1000 replicates). Red triangles indicate tick samples collected in this study.

A subset of specimens from each species was randomly selected for Sanger sequencing of the 16S rRNA, yielding a 460 bp fragment. The sequencing data have been uploaded to GenBank with login numbers PV495855 and PV495862. BLAST analysis confirmed the morphological identification for *R. microplus* and *H. longicornis*, showing 98–99% identity to corresponding tick species. Phylogenetic analysis revealed that *R. microplus* clustered into two clades, one group clustered with isolates from India, Pakistan, and Yunnan Province, while PV495855 and an isolate from Henan formed one distinct evolutionary lineage. Additionally, *H. longicornis* from Zhaotong aligned with specimens from Nanjing, Tanwai, and Jiangxi provinces, whereas *H. longicornis* from Beijing occupied distinct branches in the phylogenetic tree (Figure 3).

### 3.2. Isolation and Identification of Tick-Borne Fungi

A total of 1730 collected ticks were used for isolation and culture of fungi. A total of 90 culturable fungal isolates were obtained (Table 1), including 25 fungal species in 20 genera, with an overall isolation rate of 5.2% (90/1730). Regional fungi isolation rates varied from 0.94% to 19.21%, with Honghe showing the highest rate (19.21%), followed by Chuxiong (4.58%), while the remaining regions ranged from 0.94% to 3.30% (Table 1). In terms of quantity, a total of 40 strains were obtained from the Chuxiong region, 29 from Honghe, 12 from Kunming, and similar numbers from Zhaotong, Pu’er, and Wenshan, while the Nujiang region had the fewest, with only one strain. In terms of fungal species, ticks from Honghe yielded the richest diversity, accounting for 15 out of the 25 species identified overall, followed by Kunming and Chuxiong (eight species), whereas the remaining regions harbored only 1–2 species each. Interestingly, all 90 isolates were obtained from *R. microplus*, while no fungi were isolated from *H. longicornis*, possibly due to its limited sample size.

**Figure 4:**
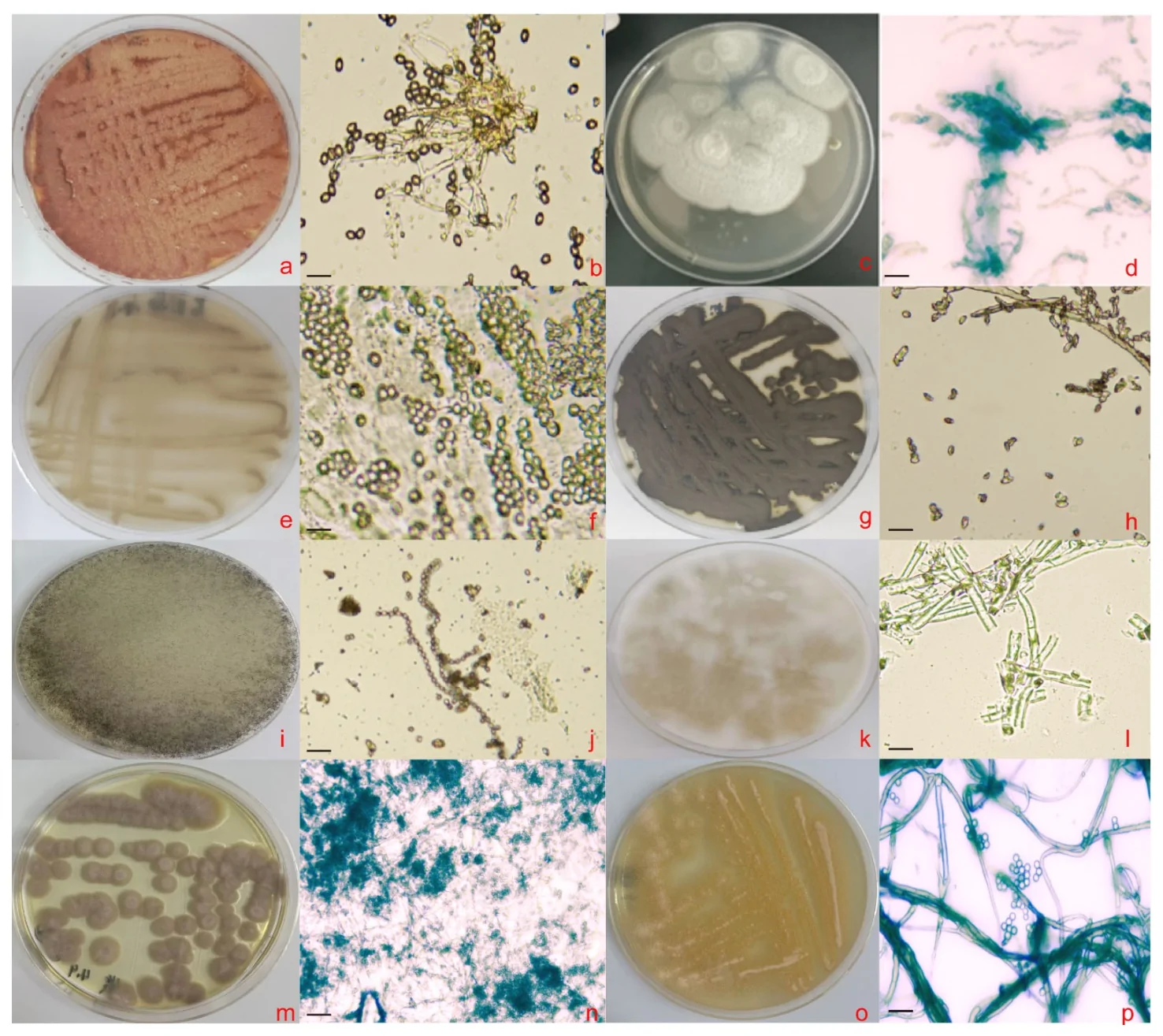
Colonial and microscopic morphology of representative fungal isolates from ticks. Scale bars: 25 μm for all microscopic panels (**b**,**d**,**f**,**h**,**j**,**l**,**n**,**p**). (**a**,**b**). *Talaromyces rufus*, (**c**,**d**). *Penicillium chermesinum*, (**e**,**f**). *Mucor bainieri*, (**g**,**h**). *Cladosporium cladosporioides*, (**i**,**j**). *Rhizopus arrhizus*, (**k**,**l**). *Schizophyllum commune*, (**m**,**n**). *Paecilomyces lilacinum*, (**o**,**p**). *Fusarium verticillioides*.

All isolates were identified based on morphological and cultural characteristics, including colony color, shape, texture, growth pattern, and microscopic features. Representative morphological characteristics of selected isolates are shown in Figure 4. On PDA, *Talaromyces marneffei* exhibited orange-red filamentous mycelium, smooth-walled conidiophores with single verticils of phialides, and smooth, globose to subglobose conidia [25]. *Penicillium chermesinum* formed gray-green, velvety colonies with typical penicilli bearing ellipsoidal to subglobose conidia. *Mucor bainieri* produced fluffy, cottony mycelium composed of broad, coenocytic hyphae, and erect sporangiophores terminated by globose to subglobose sporangia. *Cladophialophora cladosporioides* developed brownish-black, powdery colonies with septate hyphae and laterally produced conidiophores. Rhizopus arrhizus showed white, cottony mycelium bearing chains of light brown, spherical spores. *Schizophyllum commune* presented a basidiocarp with white, radially arranged gills and a trimitic hyphal system, producing cylindrical to allantoid, hyaline basidiospores [26]. *Purpureocillium lilacinum* produced lavender, floccose colonies, with conidiophores exhibiting irregular, bottleneck-shaped branches or verticils of phialides broad at the base. *Fusarium verticillioides* developed pale orange, filamentous mycelium and formed abundant, spherical microconidia on aerial hyphae.

The rDNA region of ITS sequencing of the 90 fungal isolates yielded90 distinct sequences (Table 1), including three in *Alternaria*, three in *Aspergillus*, eight in *Bjerkandera*, six in *Cladosporium*, one in *Curvularia*, eight in *Diaporthe*, one in *Eutypella*, 19 in *Fusarium*, 15 in *Geotrichum*, two in *Irpex*, two in *Rhizopus*, one in *Nigrograna*, five in *Penicillium*, one in *Phlebiopsis*, two in *Purpureocillium*, one in *Sarocladium*, eight in *Schizophyllum*, two in *Talaromyces*, one in *Mucor*, and one in *Tremateia*.

### 3.3. Taxonomic Assignation of the Culturable Tick Fungi

**Figure 5:**
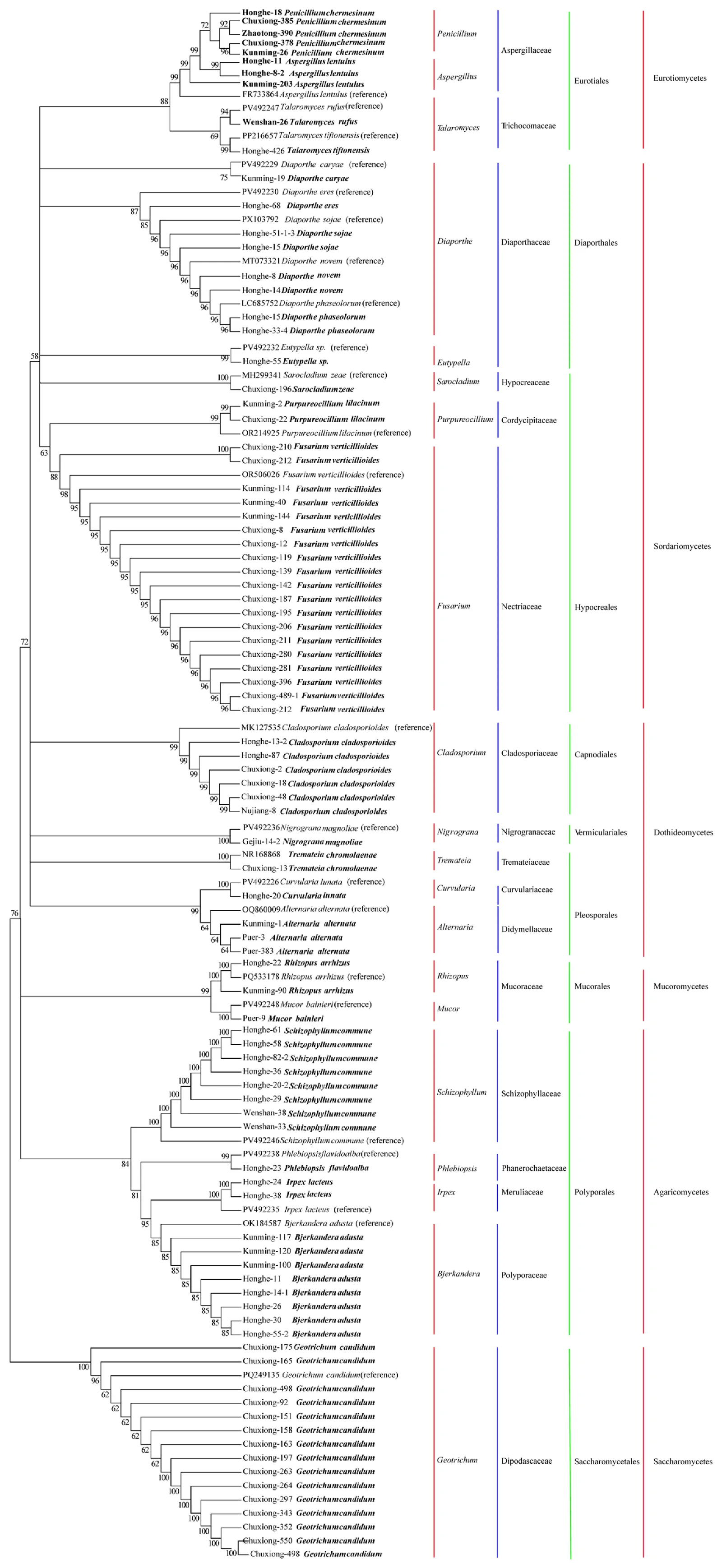
Phylogenetic tree of fungal isolates constructed using the neighbor-joining method based on ITS gene sequences, with evolutionary distances computed using the p-distance model. Bootstrap support values (1000 replicates) are shown at the nodes. Sequences obtained in this study are highlighted in bold.

**Figure 6:**
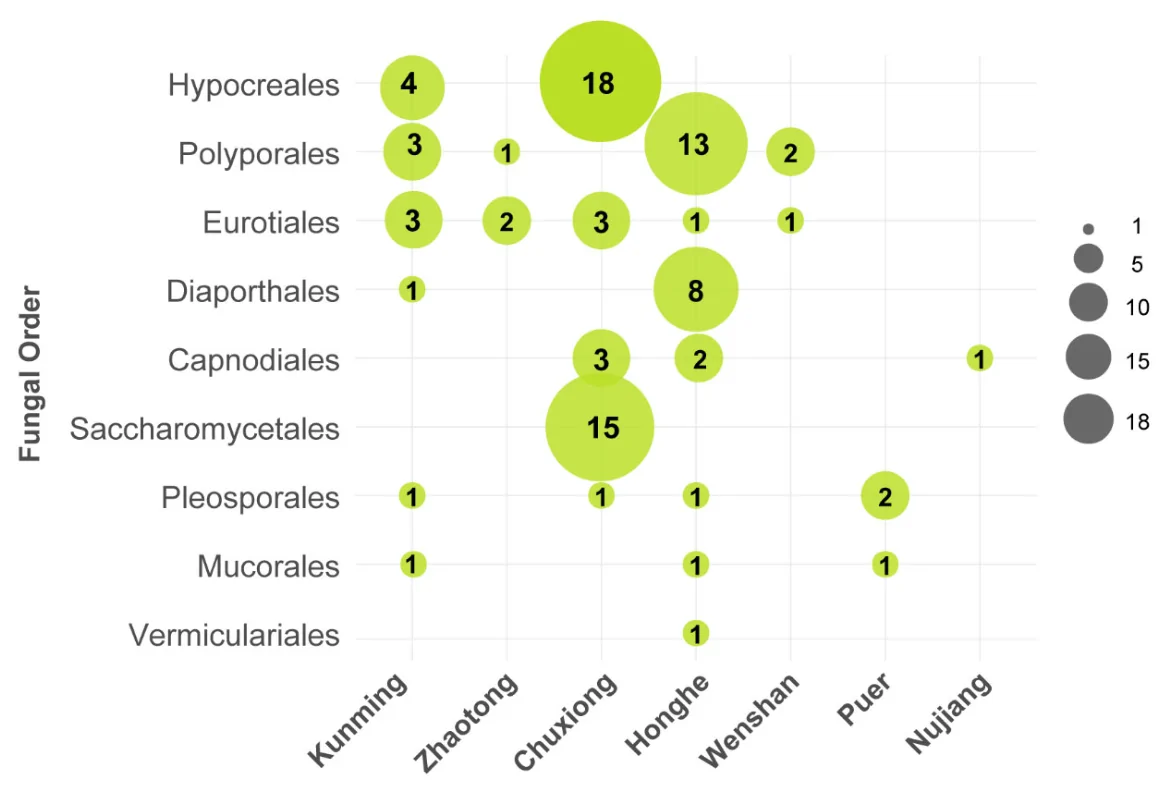
Distribution of isolates per fungal order across seven regions. Bubble size represents the number of isolates recovered per region.

The 90 fungal isolates were categorized into six divisions: Sordariomycetes (31 isolates), Agaricomycetes (19 isolates), Saccharomycetes (15 isolates), Dothideomycetes (12 isolates), Eurotiomycetes (10 isolates), and Mucoromycetes (three isolates). Based on the NJ phylogenetic analyses, all isolates were grouped into nine distinct orders (Figure 5). Within Sordariomycetes, 22 belonged to the order Hypocreales and nine to Diaporthales. In Dothideomycetes, six strains were assigned to Capnodiales, five to Pleosporales, and one to Vermiculariales. Among the classes Agaricomycetes, Eurotiomycetes, Mucoromycetes, and Saccharomycetes, each yielded isolates from only one order: 19 from Polyporales, 10 from Eurotiales, three from Mucorales, and 15 from Saccharomycetales, respectively (Figure 6).

Among these isolates, several are documented as pathogens of plants, animals, or humans. The most abundant species, *F. verticillioides* (19 isolates), is a well-known phytopathogen causing ear rot in maize and producing mycotoxins that pose risks to livestock and human health. Other plant-associated fungi included *C. cladosporioides* (six isolates) and *S. zeae* (one isolate). Notably, opportunistic animal/human pathogens such as *S. commune* (six isolates), *R. arrhizus* (two isolates), *D. phaseolorum* (two isolates), and *P. lilacinum* (two isolates) were also recovered. The presence of these fungi in ticks suggests that ticks may serve as potential vectors or environmental reservoirs for cross-kingdom pathogens. Conversely, *P. lilacinum* and *C. lunata*, as entomopathogenic fungi, hold promise as biocontrol agents against ticks, warranting further investigation.

## 4. Discussion

The survey preliminarily established the composition and distribution of tick species infesting cattle and goats in Yunnan Province. In this study, a total of 1730 tick specimens were collected from domestic animals in seven regions of Yunnan Province, including Chuxiong, Kunming, Honghe, Nujiang, Zhaotong, Pu’er, and Wenshan; 1690 (97.70%) specimens were identified as *R. microplus*, while only 40 (2.30%) were *H. longicornis*. The results revealed low species richness but wide distribution in the surveyed areas. *R. microplus* was the predominant species, which is consistent with its known high adaptability to livestock hosts and its one-host life cycle, facilitating intensive contact between susceptible animals and contaminated pastures. *R. microplus* has been proven to be one of the most frequently reported tick species in China, and it is mainly distributed in the southeastern part of the country [27,28]. In this study, it showed the highest prevalence, which is consistent with findings from many other regions of the world, including South and Central America, Africa, Asia, and Australia [29,30]. As a one-host tick, *R. microplus* completes its entire parasitic life-cycle on a single host and is considered a cattle specialist [31], though it can parasitize sheep, goats, and other wild bovids. Similarly, *H. longicornis* is also one of the most common tick species in China and is distributed widely. However, in this study, *H. longicornis* accounted for only 2.31% of the collected ticks, while *R. microplus* was overwhelmingly predominant (97.69%). This discrepancy may be attributed to differences in sampling regions, host preferences, or seasonal activity patterns, as *R. microplus* is a cattle specialist [32] while *H. longicornis* is a generalist that may be more abundant in mixed livestock–wildlife interfaces [33].

The diversity and isolation rates of culturable fungi associated with ticks varied considerably across regions in Yunnan. In this study, we obtained 90 culturable fungal isolates from 1730 ticks collected in Yunnan, representing 25 species across 20 genera with an overall isolation rate of 5.2% (90/1730). The isolation rate is lower than that reported in a recent study from France and French Guiana, where fungi were isolated from 34% of tick specimens [13]. This discrepancy may reflect differences in tick species, geographical regions, surface sterilization protocols, or culturing conditions. Notably, previous studies on *Ixodes scapularis* and *Rhipicephalus sanguineus* also reported low internal fungal prevalence (below 20%) [34], suggesting that while ticks may acquire diverse environmental fungi on their cuticle, only a limited number of fungal species can persist internally. Additionally, the isolation rate varied considerably across regions, with Honghe showing the highest rate (19.21%) and the greatest species diversity (15/25 species), suggesting that environmental conditions in this region may be particularly favorable for fungal colonization of ticks. All 90 fungal isolates were obtained exclusively from *R. microplus*, whereas no fungi were isolated from *H. longicornis*. The absence of fungal isolates from *H. longicornis* may be attributed to the limited sample size (*n* = 40) compared to *R. microplus* (*n* = 1690). Alternatively, this could reflect intrinsic biological differences between tick species, such as cuticular antimicrobial compounds or distinct microbial associations. Further studies with larger sample sizes are needed to clarify this observation.

The majority of the fungal isolates obtained in this study were documented as plant pathogens or saprophytes commonly inhabiting soil and plant surfaces. *Fusarium* was the most frequently isolated genus, with 19 strains of *F. verticillioides*, 18 of which originated from Chuxiong, suggesting a localized environmental reservoir in that area. *F. verticillioides* is a common pathogenic fungus of corn thatcauses severe yield losses and produces mycotoxins that threaten the health of both humans and livestock [35,36,37,38]. It is hypothesized that livestock are initially exposed through contaminated feed, and ticks may subsequently acquire the fungus during parasitism. The isolation of this fungus from ticks in the present study was noteworthy; however, it does not by itself confirm vector competence or tick-borne transmission. This finding highlights the necessity of further evaluating the risk of tick-borne transmission. Additionally, a diverse array of other fungal species was also isolated from tick samples, including *G. candidum* (15 isolates), *S. commune* (eight isolates), and *B. adusta* (eight isolates), as well as multiple species of Diaporthe, Alternaria, Curvularia, Penicillium, Talaromyces, and *P. lilacinum*—the latter being a well-known entomopathogenic fungus. This broad taxonomic diversity, spanning multiple phyla and ecological guilds, suggests that ticks may acquire fungi from various environmental sources, including soil, plant surfaces, and possibly even insect hosts, rather than through a single exposure route.

The isolation of opportunistic animal and human pathogens from *R. microplus* prompts consideration of their potential public health implications. Notably, *S. commune* and *R. arrhizus* are well-documented etiological agents of human respiratory allergies and mucormycosis, respectively [39,40]. Additionally, *P. lilacinum* and *D. phaseolorum*, though primarily environmental or plant-associated, have been reported to cause human endophthalmitis and ocular cutaneous infections [41,42]. Notably, the majority of other fungi isolated in this study are also commonly recovered from plants, with known pathogenicity reports for several. These are primarily plant pathogens: *C. cladosporioides* has been identified as the pathogen causing papaya scab [43], strawberry flower blight [44], and racemose blight in macadamia nuts [45]; *S. zeae* was a novel pathogen causing post-flowering stalk rot in maize in India [46]. While the vector competence of ticks for fungal pathogens remains to be experimentally demonstrated, the presence of these fungi in field-collected ticks suggests that ticks may serve as environmental reservoirs or mechanical carriers, potentially contributing to the transmission cycles of opportunistic mycoses, particularly in agricultural settings where livestock and humans are in close contact.

Ticks and tick-borne diseases (TBDs) represent a significant dual threat in Yunnan Province: they jeopardize public health and hinder the development of the livestock industry. Conversely, the isolation of entomopathogenic fungi, particularly *P. lilacinum* and *C. lunata*, presents a promising biocontrol opportunity [47]. *P. lilacinum* was documented to have efficacy against *R. microplus* under both laboratory and field conditions [48,49], highlighting its potential as a valuable biocontrol agent for integrated tick management. Additionally, several other genera isolated in this study, such as *Fusarium*, *Aspergillus*, and *Rhizopus*, have been previously documented as entomopathogenic fungi with activity against ticks [50,51]. For instance, *F. beomiforme* has demonstrated efficacy against *R. microplus*, while *A. flavus* has shown high pathogenicity against ticks [51]. Furthermore, *Rhizopus spp*. have been identified among fungal isolates capable of infecting ticks [47]. These findings suggest that the fungal diversity recovered in our study may represent a valuable reservoir for the development of novel biocontrol agents. Nevertheless, it is important to clarify that the proposed biocontrol potential of the identified entomopathogenic candidates is currently inferred from their taxonomic affiliations and previous studies, rather than from direct experimental testing performed in the present work. Therefore, subsequent studies involving standardized bioassays, dose–response assessments, and infection trials are essential to conclusively evaluate their efficacy against ticks.

Collectively, these findings paint a dual picture: ticks in Yunnan carry a diversity of fungi, including opportunistic taxa that warrant public health vigilance, alongside entomopathogenic species with promising biocontrol potential. As a first systematic survey in Yunnan, this study provides baseline data and a culture-based inventory of fungal associates. Meanwhile, we recognize that culture-dependent methods may not capture the full fungal diversity, and that functional experiments, such as vector competence tests, are needed to clarify the transmission potential of fungi isolated. Our findings thus lay a foundation for future studies to build upon, particularly in assessing the ecological roles of these fungi and their translational values.

## 5. Conclusions

In conclusion, this study systematically investigated the distribution of parasitic ticks infesting livestock in Yunnan Province and the culturable fungi associated with them. *R*. *microplus* was identified as the predominant tick species across the surveyed regions, and a total of 90 fungal strains representing 25 species across 20 genera were isolated, with *F. verticillioides* being the most prevalent species. The recovery of both opportunistic taxa and entomopathogenic fungi from ticks highlights the dual significance of this investigation: it raises awareness of the fungal diversity inticks and identifies promising candidates for biological control. These findings provide a foundational basis for future functional studies, particularly vector competence assessments, to clarify the epidemiological significance of tick–fungus associations and to inform integrated tick management in this region.

## Figures and Tables

**Table 1:** Fungal isolates from ticks.

Classification	Species Name	Number of Strains							
		Kunming (*n* = 345)	Honghe (*n* = 151)	Chuxiong (*n* = 874)	Zhaotong (*n* = 80)	Pu’er (*n* = 91)	Nujiang (*n* = 106)	Wenshan (*n* = 83)	Total (*n* = 1730)
*Alternaria*	*Alternaria alternata*	1				2			3
*Aspergillus*	*Aspergillus lentulus*	2			1				3
*Bjerkandera*	*Bjerkandera adusta*	3	5						8
*Cladosporium*	*Cladosporium cladosporioides*		2	3			1		6
*Curvularia*	*Curvularia lunata*		1						1
*Diaporthe*	*Diaporthe novem*		2						8
	*Diaporthe phaseolorum*		2						
	*Diaporthe caryae*	1							
	*Diaporthe eres*		1						
	*Diaporthe sojae*		2						
*Eutypella*	*Eutypella* sp.		1						1
*Fusarium*	*Fusarium verticillioides*	3		16					19
*Geotrichum*	*Geotrichum candidum*			15					15
*Irpex*	*Irpex lacteus*		1		1				2
*Rhizopus*	*Rhizopus arrhizus*	1	1						2
*Nigrograna*	*Nigrograna magnoliae*		1						1
*Penicillium*	*Penicillium chermesinum*	1	1	2	1				5
*Phlebiopsis*	*Phlebiopsis flavidoalba*		1						1
*Purpureocillium*	*Purpureocillium lilacinum*	1		1					2
*Sarocladium*	*Sarocladium zeae*			1					1
*Schizophyllum*	*Schizophyllum commune*		6					2	8
*Talaromyces*	*Talaromyces tiftonensis*			1					2
	*Talaromyces rufus*							1	
*Mucor*	*Mucor bainieri*					1			1
*Tremateia*	*Tremateia chromolaenae*			1					1
Total		12	29	40	2	3	1	3	90
Isolation rate (%)		1.37	19.21	4.58	2.5	3.30	0.94	2.83	5.20

## Data Availability

The original contributions presented in this study are included in the article. Further inquiries can be directed to the corresponding author.

## References

[1] Kebzai F., Ashraf K., Rehman M.U., Akbar H., Avais M. (2024). Prevalence and associated risk factors of ixodid tick species infesting cattle and sheep in Balochistan, Pakistan. Vet. Parasitol. Reg. Stud. Rep..

[2] Zhang Y.K., Zhang X.Y., Liu J.Z. (2019). Ticks (Acari: Ixodoidea) in China: Geographical distribution, host diversity, and specificity. Arch. Insect Biochem. Physiol..

[3] Zhang K., Li A., Wang Y., Zhang J., Chen Y., Wang H., Shi R., Qiu Y. (2021). Investigation of the presence of *Ochrobactrum* spp. and *Brucella* spp. in *Haemaphysalis longicornis*. Ticks Tick. Borne Dis..

[4] Sonenshine D.E. (2018). Range Expansion of Tick Disease Vectors in North America: Implications for Spread of Tick-Borne Disease. Int. J. Environ. Res. Public Health.

[5] Zhong Z., Wang K., Wang J. (2024). Tick symbiosis. Curr. Opin. Insect Sci..

[6] Ajileye O.D., Verocai G.G., Light J.E. (2025). A review of filarial nematodes parasitizing tick vectors: Unraveling global patterns in species diversity, host associations, and interactions with tick-borne pathogens. Parasit. Vectors.

[7] Mollong E., Lébri M., Marie-Magdeleine C., Lagou S.M., Naves M., Bambou J.C. (2025). Sustainable management of tick infestations in cattle: A tropical perspective. Parasit. Vectors.

[8] Nava S., Rossner M.V., Toffaletti J.R., Da Luz M., Rossner M.B., Signorini M., Morel N. (2024). Strategic control of the cattle tick *Rhipicephlaus microplus* applied to rotational and silvopastoral grazing systems in subtropical areas. Parasitol. Res..

[9] Bonnet S.I., Binetruy F., Hernández-Jarguín A.M., Duron O. (2017). The Tick Microbiome: Why Non-pathogenic Microorganisms Matter in Tick Biology and Pathogen Transmission. Front. Cell Infect. Microbiol..

[10] Bonnet S.I., Pollet T. (2021). Update on the intricate tango between tick microbiomes and tick-borne pathogens. Parasite Immunol..

[11] Duron O., Gottlieb Y. (2020). Convergence of Nutritional Symbioses in Obligate Blood Feeders. Trends Parasitol..

[12] Perinotto W.M., Angelo I.C., Golo P.S., Quinelato S., Camargo M.G., Sá F.A., Bittencourt V.R. (2012). Susceptibility of different populations of ticks to entomopathogenic fungi. Exp. Parasitol..

[13] Bruley M., Pasternicki C., Fattar N., Amoros J., Duhayon M., McCoy K., Duron O. (2025). Culturable bacteria and fungi in Ixodes, Dermacentor, Amblyomma and Ornithodoros ticks. Parasite.

[14] Clay K., Klyachko O., Grindle N., Civitello D., Oleske D., Fuqua C. (2008). Microbial communities and interactions in the lone star tick, *Amblyomma americanum*. Mol. Ecol..

[15] Narasimhan S., Fikrig E. (2015). Tick microbiome: The force within. Trends Parasitol..

[16] Narasimhan S., Swei A., Abouneameh S., Pal U., Pedra J.H.F., Fikrig E. (2021). Grappling with the tick microbiome. Trends Parasitol..

[17] Hayes B.M., Radkov A.D., Yarza F., Flores S., Kim J., Zhao Z., Lexa K.W., Marnin L., Biboy J., Bowcut V. (2020). Ticks Resist Skin Commensals with Immune Factor of Bacterial Origin. Cell.

[18] Du C.H., Yang J.H., Yao M.G., Jiang B.G., Zhang Y., He Z.H., Xiang R., Shao Z.T., Luo C.F., Pu E.N. (2024). Systematic investigation of the Borrelia miyamotoi spirochetes in ticks, wildlife and domestic animal hosts in Yunnan province, Southwest China. One Health.

[19] Chen Z., Liu J.-Z. (2020). Recent progress in tick taxonomy and a global list of tick species. Chin. J. Appl. Entomol..

[20] Tidwell J.P., Treviño D.E., Thomas D.B., Mitchell R.D., Heerman M.C., Pérez de León A., Lohmeyer K.H. (2021). Pictorial dissection guide and internal anatomy of the cattle tick, *Rhipicephalus* (*Boophilus*) *microplus* (Canestrini). Ticks Tick. Borne Dis..

[21] Barra P., Rosso L., Nesci A., Etcheverry M. (2013). Isolation and identification of entomopathogenic fungi and their evaluation against *Tribolium confusum*, *Sitophilus zeamais*, and *Rhyzopertha dominica* in stored maize. J. Pest Sci..

[22] Chen Z., Li Y., Ren Q., Luo J., Liu Z., Zhou X., Liu G., Luo J., Yin H. (2014). *Dermacentor everestianus* Hirst, 1926 (Acari: Ixodidae): Phylogenetic status inferred from molecular characteristics. Parasitol. Res..

[23] Crous P., Schumacher R., Akulov A., Thangavel R., Hernández-Restrepo M., Carnegie A., Cheewangkoon R., Wingfield M., Summerell B., Quaedvlieg W. (2019). New and interesting fungi. 2. Fungal Syst. Evol..

[24] Xu J. (2016). Fungal DNA barcoding. Genome.

[25] Barbosa R.N., Bezerra J.D.P., Souza-Motta C.M., Frisvad J.C., Samson R.A., Oliveira N.T., Houbraken J. (2018). New Penicillium and Talaromyces species from honey, pollen and nests of stingless bees. Antonie Leeuwenhoek.

[26] Dasgupta D., Basu P., Paul A., Acharya K., Chakraborty N. (2025). *Schizophyllum commune*, an underrated edible and medicinal mushroom: Farm to industry. Stud. Fungi.

[27] Zhang G., Zheng D., Tian Y., Li S. (2019). A dataset of distribution and diversity of ticks in China. Sci. Data.

[28] Xia H., Hu C., Zhang D., Tang S., Zhang Z., Kou Z., Fan Z., Bente D., Zeng C., Li T. (2015). Metagenomic profile of the viral communities in Rhipicephalus spp. ticks from Yunnan, China. PLoS ONE.

[29] Antas P.R., Brito M.M., Peixoto E., Ponte C.G., Borba C.M. (2012). Neglected and emerging fungal infections: Review of hyalohyphomycosis by *Paecilomyces lilacinus* focusing in disease burden, in vitro antifungal susceptibility and management. Microbes Infect..

[30] Kanduma E.G., Emery D., Githaka N.W., Nguu E.K., Bishop R.P., Šlapeta J. (2020). Molecular evidence confirms occurrence of *Rhipicephalus microplus* Clade A in Kenya and sub-Saharan Africa. Parasit. Vectors.

[31] McCoy K.D., Léger E., Dietrich M. (2013). Host specialization in ticks and transmission of tick-borne diseases: A review. Front. Cell Infect. Microbiol..

[32] Johnson N. (2023). Chapter 3—The tick life cycle. Ticks.

[33] Butler R.A., Muller L.I., Grove D., Fryxell R.T.T. (2024). Ecological relationships of *Haemaphysalis longicornis* Neumann with other tick species on wildlife hosts at cow–calf farms implementing integrated pest management in eastern Tennessee. Parasitology.

[34] Benoit J.B., Yoder J.A., Ark J.T., Rellinger E.J. (2005). Fungal fauna of *Ixodes scapularis* say and *Rhipicephalus sanguineus* (Latreille) (Acari: Ixodida) with special reference to species-associated internal mycoflora. Int. J. Acarol..

[35] Xu Y., Zhang Z., Lu P., Li R., Ma P., Wu J., Li T., Zhang H. (2023). Increasing *Fusarium verticillioides* resistance in maize by genomicsassisted breeding: Methods, progress, and prospects. Crop J..

[36] Ding Y., Ma N., Haseeb H.A., Dai Z., Zhang J., Guo W. (2024). Genome-wide transcriptome analysis of toxigenic *Fusarium verticillioides* in response to variation of temperature and water activity on maize kernels. Int. J. Food Microbiol..

[37] Missmer S.A., Suarez L., Felkner M., Wang E., Merrill A.H., Rothman K.J., Hendricks K.A. (2006). Exposure to fumonisins and the occurrence of neural tube defects along the Texas-Mexico border. Environ. Health Perspect..

[38] Gelineau-van Waes J., Starr L., Maddox J., Aleman F., Voss K.A., Wilberding J., Riley R.T. (2005). Maternal fumonisin exposure and risk for neural tube defects: Mechanisms in an in vivo mouse model. Birth Defects Res. A Clin. Mol. Teratol..

[39] Chowdhary A., Randhawa H.S., Gaur S.N., Agarwal K., Kathuria S., Roy P., Klaassen C.H., Meis J.F. (2013). *Schizophyllum commune* as an emerging fungal pathogen: A review and report of two cases. Mycoses.

[40] Martinello M., Nelson A., Bignold L., Shaw D. (2012). “We are what we eat!” Invasive intestinal mucormycosis: A case report and review of the literature. Med. Mycol. Case Rep..

[41] Luangsa-Ard J., Houbraken J., van Doorn T., Hong S.B., Borman A.M., Hywel-Jones N.L., Samson R.A. (2011). *Purpureocillium*, a new genus for the medically important *Paecilomyces lilacinus*. FEMS Microbiol. Lett..

[42] Mattei A.S., Severo C.B., Guazzelli L.S., Oliveira F.M., Gené J., Guarro J., Cano J., Severo L.C. (2013). Cutaneous infection by *Diaporthe phaseolorum* in Brazil. Med. Mycol. Case Rep..

[43] Chen R.S., Wang W.L., Li J.C., Wang Y.Y., Tsay J.G. (2009). First Report of Papaya Scab Caused by *Cladosporium cladosporioides* in Taiwan. Plant Dis..

[44] Nam M.H., Park M.S., Kim H.S., Kim T.I., Kim H.G. (2015). *Cladosporium cladosporioides* and *C. tenuissimum* Cause Blossom Blight in Strawberry in Korea. Mycobiology.

[45] van den Berg N., Serfontein S., Christie B., Munro C. (2008). First Report of Raceme Blight Caused by *Cladosporium cladosporioides* on Macadamia Nuts in South Africa. Plant Dis..

[46] Harish J., Prasannakumar M.K., Venkateshbabu G., Karan R., Mahesh H.B., Devanna P., Sarangi A.N., Patil S.S., Tejashwini V., Lohithaswa H.C. (2025). Molecular and genomic insights into the pathogenicity of *Sarocladium zeae* causing maize stalk rot disease. Microbiol. Res..

[47] Casasolas-Oliver A., Estrada-Pena A., Gonzalez-Cabo J., Dusbadek E., Bukva V. (1991). Activity of *Rhizopus thailandensis*, *Rhizopus arrhizus* and *Curvularia lunata* on reproductive efficacy of *Rhipicephalus sanguineus* (Ixodidae). Modern Acaralogy.

[48] Pereira J.R., Nicodemos F.G., Duarte F.C., de Almeida J.E.M., Mendes M.C. (2024). Efficacy of the fungus *Purpureocillium lilacinum* applied via drone onto pasture for controlling the tick *Rhipicephalus* (*Boophilus*) *microplus* (Acari: Ixodidae). Vet. Parasitol..

[49] Angelo I.C., Fernandes É.K., Bahiense T.C., Perinotto W.M., Golo P.S., Moraes A.P., Bittencourt V.R. (2012). Virulence of *Isaria* sp. and *Purpureocillium lilacinum* to *Rhipicephalus microplus* tick under laboratory conditions. Parasitol. Res..

[50] Ebani V.V., Mancianti F. (2021). Entomopathogenic Fungi and Bacteria in a Veterinary Perspective. Biology.

[51] Arreguin-Perez C.A., Miranda-Miranda E., Folch-Mallol J.L., Cossío-Bayúgar R. (2023). Identification of Virulence Factors in Entomopathogenic *Aspergillus* flavus Isolated from Naturally Infected *Rhipicephalus microplus*. Microorganisms.

